# Experience with impacted upper ureteral Stones; should we abandon using semirigid ureteroscopes and pneumatic lithoclast?

**DOI:** 10.1186/1755-7682-2-13

**Published:** 2009-05-03

**Authors:** Ehab ElGanainy, Diaa A Hameed, MA Elgammal, Alaa A Abd-Elsayed, M Shalaby

**Affiliations:** 1 Urology Department, Assiut University Hospital, Assiut, Egypt; 2 Public health and Community Medicine Department, Faculty of medicine, Assiut University, Assiut, Egypt

## Abstract

**Introduction:**

The AUA/EAU Ureteral Stones Guideline Panel reported that the stone free rate for the proximal ureteral stones is around 81% when treated by either SWL or ureteroscopy (URS).

Complication rates, most notably ureteral perforation and long-term complications of URS such as stricture formation rates, have been reduced to < 5%. Moreover, impacted ureteral calculi are more difficult to fragment with SWL because of the lack of natural expansion space for stones, this result in a situation that is better managed by ureteroscopy. The aim of this study is to assess the efficacy, safety, and complications of impacted upper ureteral stone disintegration using semirigid ureteroscopes and pneumatic lithotripsy.

**Methods:**

We retrospectively analyzed the records of 267 consecutive patients with impacted upper ureteral stones (9–20 mm) who were treated by semirigid ureteroscopes and pneumatic disintegration. The efficacy of treatment was estimated using the stone-free rate and all treatment related complications were analyzed.

**Results:**

Except for 24 cases where the stone migrated to the kidney, the stone was successfully treated ureteroscopically, with a low rate of minimal complications such as mild hematuria (18.4%), short term low grade fever (13.5%). Only 3 patients (1.1%) had high grade fever and none had post operative stricture.

**Conclusion:**

The use of semirigid URS and pneumatic lithotripsy in impacted upper ureteral stones in experienced hands has very satisfactory results with minimal complications. When Holmium laser and flexible URS are not available, semirigid URS and pneumatic lithotripsy is a good alternative that shouldn't, yet, be abandoned.

## Introduction

In the early 1980s, the development of shock wave lithotripsy (SWL) changed the therapeutic modalities for urinary stones [1]. The AUA/EAU Ureteral Stones Guideline Panel reported that the stone free rate for both SWL and ureteroscopy (URS) when treating proximal ureteral stones is around 81%. The rate for stones >10 mm decreased to 68% and 79% if they were treated by SWL and URS respectively [2].

URS has traditionally constituted the favored approach for the surgical treatment of mid and distal ureteral stones while SWL has been preferred for the less accessible proximal ureteral stones. With the development of smaller caliber semirigid and flexible ureteroscopes and the introduction of improved instrumentation, including the holmium: YAG laser, URS has evolved into a safer and more efficacious modality for treatment of stones in all locations in the ureter with increasing experience worldwide [3,4]. Complication rates, most notably ureteral perforation rates, have been reduced to less than 5%, and long-term complications such as stricture formation occur with an incidence of 2% or less. Overall stone-free rates are remarkably high at 81% to 94% depending on stone location, with the vast majority of patients rendered stone free in a single procedure [2].

Moreover, impacted ureteral calculi are more difficult to fragment with SWL because of the lack of natural expansion space for the stones in the ureter, this result in a situation that is better managed by ureteroscopy [5-7]. Advances in endoscope design and the development of intracorporeal lithotripsy devices such as the Swiss lithoclast and holmium:YAG laser made the endoscopic treatment of any ureteral stone a possibility [8-10].

In this study we aimed at evaluation of the results of our management of the impacted upper ureteral stones using the semirigid ureteroscopes and pneumatic lithotripsy.

## Patients and methods

Retrospective analysis of the data of 267 patients (218 males and 49 females; age range 18 to 69) who presented to urology department, Assiut university hospital, between April 2001 and April 2007 complaining of loin pain due to impacted upper ureteral stones (severely adherent or causing sever ureteral wall edema) was done. The patients had previous medical treatment for more than 1 month and 56 of them had at least one failed trial of SWL.

Among the patients, 6 were 24–28 weeks pregnant females (who preferred ureteroscopy than percutaneous nephrostomy for such a long period till delivery), and 10 showed liver impairment (prothrombin concentration below 60%). The pregnant females were evaluated by US and MRU.

The patients had a single stone in 236 (88.4%) and multiple stones in 31 cases (2 stones in 30 and 3 stones in one). The stone length ranged from 9 to 20 mm with a mean of 12.4 mm (70% <15 mm and 30% ≥ 15 mm); all were impacted in the upper ureter; 12 stones (4.5%) opposite L2, 20 (7.5%) opposite L3, 75 stones (28%) opposite L4 and 160 stones (60%) opposite L5.

All patients had spinal anesthesia (except 9 patients who had general anesthesia), were put in a mild telendenberg's position then given IV furesamide 40 mg to guard against stone migration after disimpaction during pneumatic disintegration.

The operation started by identification of the ureteral orifice and retrograde ureterography. A trial to gently advance a safety floppy tip 0.035 inch guide wire past the stone was done. Whether the wire passed the stone or not, we dilated of the ureteral orifice till 12 ch. then URS using R. Wolf long ureteroscope (8.5 ch. Tip) was done until the stone was reached.

Disintegration using the Swiss pneumatic lithoclast was done and the stone gravels were retrieved using Dormia basket, but in case of smaller gravels a grasper was used to ensure removal of all sizable gravels. At the end of the maneuver ureterography is done to exclude perforation. In case of the pregnant females, x ray wasn't used.

A DJ stent was applied for 6 weeks (until delivery in the pregnant females). Three months after its removal, x ray and ultrasound were done to ensure the stone free state and to evaluate the resolution of hydronephrosis. Success was defined as having no stone gravels visible on x ray or US after removal of the DJ, a non obstructed kidney and no symptoms 3 months after DJ removal.

## Results

No patient was anuric or oliguric, all had normal chemistry with non obstructed contralateral renal unit. IVU showed epsilateral hydronephrosis ranging from 1^st ^to 4^th ^degree (68 patients had 1^st ^degree hydronephrosis, 160 had 2^nd^, 35 had 3^rd ^and 4 patients had 4^th ^degree) with no contrast media passing the stone.

Operative time ranged from 38 to 62 min (mean 55 min), table 1. Stones or stone gravels migrated to the kidney in 24 cases (~9%) during trials of disintegration. These cases were managed by applying a DJ stent and the patient was sent to the SWL unit. The dormia basket was impacted during stone retrieval in 19 cases (7.12%) due to the unexpected large size of the retrieved stone gravels, the handle of the basket was unscrewed and the ureteroscope removed and reintroduced alongside the dormia wires and disintegration continued, then the smaller size gravels were removed as usual.

**Table 1:** The effect of multiplicity, laterality, stone length and gender on stone free rate, operative time, extravasation and hematuria.

		**Multiplicity**		**Laterality**		**Stone length (mm)**		**Sex**	
	Total	Single 236	Multiple 31	Rt 159	Lt 108	<15 187	≥ 15 80	Male 218	Female 49

Stone free rate	91% (n = 243)	215	28 (*p *= 0.85)*	143	100 (*p *= 0.46)*	167	76 (*p *= 0.21)*	196	47 (*p *= 0.27)*

Operative time (minutes)	Mean 55	54.1 ± 6.1	55.9 ± 6.5 (*p *= 0.13)*	54.6 ± 5.1	55.6 ± 5.5 (*p *= 0.13)*	54.1 ± 5.1	55.9 ± 5.5 (*p *= 0.01)^$^	55.8 ±5.9	54 ± 6.1 (*p *= 0.07)*

Extravasation	6.7% (n = 18)	15	3 (*p *= 0.45)*	10	8 (*p *= 0.72)*	12	6 (*p *= 0.75)*	16	2 (*p *= 0.54)*

Hematuria	18.4% (n = 49)	43	6 (*p *= 0.88)*	34	15 (*p *= 0.12)*	38	11 (*p *= 0.2)*	41	8 (*p *= 0.69)*

* *p *value > 0.05 (insignificant).

^$^*p *value is < 0.05 (significant).

The multiplicity, laterality and size of the stones didn't make a significant difference regarding the stone free rate or retropulsion rate, table 1.

There was statistically significant positive correlation between the operative time and the stone size (p < 0.01 & r = 0.865). The female gender had slightly higher success rate, shorter operative time, yet this wasn't statistically significant, table 1.

There were no serious operative or post operative complications. Minor extravasation was found at the end of the maneuver in 18 cases (6.7%), table 1, but URS didn't reveal a significant perforation and DJ stent was applied as usual. Mild hematuria was encountered in 49 patients (18.4%) that lasted for less than 48 hours and treated conservatively, table 1. Low grade fever (38°C) was encountered in 36 patients (13.5%), lasted for <48 hours and was managed by broad spectrum antibiotics and oral antipyretics. High grade fever occurred in 3 of 4 cases that had 4^th ^degree hydronephrosis, urine culture was done and antibiotic treatment was given accordingly and fever subsided within 5 days.

One hundred and four patients (39%) were available for follow up 3 months or later after removal of the DJ. Plain X ray and ultrasound were done. No residual stones were detected. The kidneys with degrees 1: 2 of hydronephrosis regained their normal shape (91 patients), while those with more sever degrees retained some degree of residual dilatation of the pelvicalyceal system which was asymptomatic (13 patients).

## Discussion

In this study the records of 267 patients with impacted upper ureteral stones treated by URS and pneumatic disintegration were reviewed. Stone free rate reached 91% with low rate of minor complications.

There are several definitions of impacted ureteral stone. Morgantaler definition of it is that the doctor can not make a guide wire pass the stone [11]. Srivastava considered that the stone should cause moderate or severe hydronephrosis [12]. Deliveliotis defined it as the stone that stays in the same place of the upper ureter for at least 2 months [13]. Because two thirds of all stones that passes spontaneously do so within 4 weeks [14], we used the term impacted for those stones that stay in the same position for more than one month, causes symptoms and that don't allow contrast past them in the IVU. The rationale is that patients usually can't wait longer before intervention takes place because of pain and agitation about having a stone that doesn't seem to be passing down. On doing URS those who had a stone that is adherent to ureteral wall or causing sever edema are documented as impacted stones and were included in this study.

Although SWL is the least invasive and an effective modality of treatment of upper ureteral calculi, yet this isn't the case for impacted stones. Ureteroscopic management achieves a similar or higher success rates than SWL, faster stone delivery and instant resolution of the obstruction [15].

In this study we treated 267 patients with impacted upper ureteral calculi causing different degrees of hydronephrosis by pneumatic disintegration and dormia extraction.

The mean operative time was 55 min. The stone free rate reached 91%, the failures were due to retropulsion of the stone or its gravels (9%). In female patients mean operative time was slightly shorter and stone free rate was slightly more than that in the male patients, this is due to the shorter female urethra, hence the easiness of introduction of the ureteroscope and access to the stones especially those just below the UPJ.

In the present study successful disintegration and retrieval of the stone occurred in 91% of cases, retropulsion occurred in 9%. Stone retropulsion during disintegration of upper ureteral calculi can occur even when using Holmium laser. In a study on 208 cases of ureteral stones, 55 of them upper ureteral, Gupta reported a 3.3% failure rate due to retropulsion using Holmium laser [16]. We believe that in the case of impacted stones and hydronephrosis, stone disimpaction, ureteral stenting and later SWL, which is done in this study if reteropulsion occurred, gives the patient the benefit of instantaneous pain relief as there is no more obstruction and SWL for a stone 9–20 mm in the kidney is usually a successful task.

Many reports of similar studies promote the use of semirigid ureteroscopes in treatment of upper ureteral calculi including large and impacted stones [17-21]. Yet other reports express concerns about the stone free rate [22], and complications of the procedure [23].

Harmon et al. reported the rate of stricture formation after URS to be 0.5% in 1992 compared to 1.5% 10 years earlier [24]. In our study there was no stricture among patients available for follow up. The application of better ureteroscopes and viewing armamentarium besides the increasing experience with the procedure certainly decrease the complication rate. This low stricture rate is now common in the more recent reports [22]. The minor complications that occurred in our patients were all managed conservatively and had no long term consequences. The minor perforations that occurred didn't prevent the passage of the ureteroscope or application of the DJ, these perforations may occur due to the edema and fragility of the mucosa, the disintegration process or due to the trial of advancement of the dormia basket past the stone gravels.

Hematuria due to mucosal abrasion or minor perforation was mild and stopped within 48 hours of conservation. Post operative low grade fever occurred in 36 patients and high grade fever in 3 patients, the latter had more advanced hydronephrosis, that could make kidney drainage by a DJ less effective, this stagnation can invite infection after a ureteroscopic procedure. Yet, all the patients responded to conservative treatment as there was no need for further drainage of the kidney although removal of the urethral catheter had to be postponed for one day after subsidence of the fever.

## Conclusion

The use of semirigid URS and pneumatic lithotripsy in impacted upper ureteral stones in experienced hands has very satisfactory results with minimal complications. When Holmium laser and flexible URS are not available, semirigid URS and pneumatic lithotripsy is a good alternative that shouldn't, yet, be abandoned.

## Competing interests

The authors declare that they have no competing interests.

## Authors' contributions

EEG, DAH, MAE, AAA-E and MS carried out the patient care, investigation, management, follow up, drafting the manuscript and writing the final version.

All authors read and approved the final manuscript.
